# 907. Resilience Amidst Disparity: Family Income and Recovery Outcomes in Pediatric Burn Survivors

**DOI:** 10.1093/jbcr/irag033.411

**Published:** 2026-04-06

**Authors:** Sarah Wang, Trevor A Pickering, Shelley A Wiechman, Kimberly Roaten, Colleen M Ryan, Kara McMullen, Haig A Yenikomshian

**Affiliations:** Keck School of Medicine, University of Southern California, Los Angeles, CA; Department of Population and Public Health Sciences, Keck School of Medicine, University of Southern California, Los Angeles, CA; University of Washington Harborview Medical Center, Seattle, WA; Department of Psychiatry, University of Texas Southwestern Medical Center, Dallas, TX; Massachusetts General Hospital, Harvard Medical School, Boston, MA; Department of Rehabilitation Medicine, University of Washington, Seattle, WA; Division of Plastic and Reconstructive Surgery, Keck School of Medicine, University of Southern California, Los Angeles, CA

## Abstract

**Introduction:**

Pediatric burn survivors often demonstrate remarkable resilience, especially when supported by family and with consistent access to care. However, children from lower income families are disproportionately affected by burns, experiencing more severe injuries and potentially greater psychosocial challenges. This study examines whether family income influences the ability of pediatric burn patients to return to baseline function.

**Methods:**

A retrospective study was conducted with data that was collected from a multi-center longitudinal database between 2015 and 2024. Participants included pediatric individuals (< 18) with family income data. There were three cohorts of patients based on income level: <$50 k, $50 k - $100 k, and > $100 k. Return to school outcomes and psychosocial outcomes (PROMIS Depression, PROMIS Anger, PROMIS Peer Relationships, PROMIS Anxiety, PROMIS Pain Interference, PROMIS Family Relationships, NIH Anger, NIH Sadness, NIH General Life Satisfaction, Post-Traumatic Growth, Child PTSD Scale, Body Image, and Pain Intensity) were collected at 12 months after injury from self-report by individuals ages 8-17. Cohorts were compared using Kruskal-Wallis tests and regression models which adjusted for %TBSA and inhalation injury, with a significance set at *p*<.05.

**Results:**

The final analytic sample of participants with family income data consisted of 115 individuals (56.4%) with income <$50 k, 55 (27.0%) with income $50 k - $100 k, and 34 (17.4%) with income >$100 k. The majority of patients were male (n = 125, 61.3%). On average, the % TBSA burned was significantly higher (*p*<.001) and there was a greater incidence of inhalation injury (*p*=.03) in children with a family income of <50 k. Participants in this cohort over the age of 7 had greater post-traumatic growth than the other two groups (*p*<.05). Children in the lowest income group scored higher on the PROMIS pain interference scale at follow-up (*p*=.02), indicating their pain made it more difficult to carry out daily activities. However, this was no longer significant after adjusting for % TBSA burned (*p*=.31). While family income levels showed no association with differences in time to return to school (*p*=.40), greater burn size significantly delayed recovery (*p*=.02).

**Conclusions:**

Lower family income level was not significantly associated with worse psychosocial outcomes but did correlate to more severe burn injuries. These findings show that lower socioeconomic status does not necessarily impede recovery in pediatric burn survivors; however, there is a need to address systemic inequalities that contribute to the disproportionate injury burden faced by children from lower income families.

**Applicability of Research to Practice:**

Providers should proactively allocate resources for rehabilitation to children from lower income families, who are at the highest risk for severe burn injuries.

**Funding for the study:**

The contents of this abstract were developed under a grant from the National Institute on Disability, Independent Living, and Rehabilitation Research (NIDILRR grant number 90DPBU0007). NIDILRR is a Center within the Administration for Community Living (ACL), Department of health and Human Services (HHS). The contents of this abstract do not necessarily represent the policy of NIDILRR, ACL, or HHS, and you should not assume endorsement by the Federal Government.

